# Discovering charge density functionals and structure-property relationships with PROPhet: A general framework for coupling machine learning and first-principles methods

**DOI:** 10.1038/s41598-017-01251-z

**Published:** 2017-04-26

**Authors:** Brian Kolb, Levi C. Lentz, Alexie M. Kolpak

**Affiliations:** 1 Massachusetts Institute of Technology, Mechanical Engineering, Cambridge, MA 02139 USA; 2 University of New Mexico, Department of Chemistry and Chemical Biology, Albuquerque, NM 87110 Mexico

## Abstract

Modern *ab initio* methods have rapidly increased our understanding of solid state materials properties, chemical reactions, and the quantum interactions between atoms. However, poor scaling often renders direct *ab initio* calculations intractable for large or complex systems. There are two obvious avenues through which to remedy this problem: (i) develop new, less expensive methods to calculate system properties, or (ii) make existing methods faster. This paper describes an open source framework designed to pursue both of these avenues. PROPhet (short for PROPerty Prophet) utilizes machine learning techniques to find complex, non-linear mappings between sets of material or system properties. The result is a single code capable of learning analytical potentials, non-linear density functionals, and other structure-property or property-property relationships. These capabilities enable highly accurate mesoscopic simulations, facilitate computation of expensive properties, and enable the development of predictive models for systematic materials design and optimization. This work explores the coupling of machine learning to *ab initio* methods through means both familiar (e.g., the creation of various potentials and energy functionals) and less familiar (e.g., the creation of density functionals for arbitrary properties), serving both to demonstrate PROPhet’s ability to create exciting post-processing analysis tools and to open the door to improving *ab initio* methods themselves with these powerful machine learning techniques.

## Introduction

Recent decades have seen a surge in the development of first principles methods aimed at increasing our understanding of the interactions of atoms in materials and other chemical systems. In addition to total energy calculations with the workhorse density functional theory^1, 2^ (DFT), advanced methods now allow unprecedented accuracy in computations of wide ranging material properties of practical interest, including optical properties (GW^3^, TD-DFT^4^, and BSE^5^), thermal and electronic transport characteristics (Boltzmann transport based methods), response properties (perturbative methods), electronic polarization (the Berry phase formalism^6^), and many others. These methods, however, often have a computational cost orders-of-magnitude greater than a simple ground-state total energy calculation. The expense of computing these important properties (or even just extremely accurate total energies computed using high-order methods such as CCSD(T)^7^) can be prohibitive for many important systems. The development of ever more sophisticated materials, increasingly requiring a detailed treatment of interfaces, heterostructures, and environmental factors, brings with it a concomitant need for advanced methods capable of treating these large, complex systems.

In this work, we describe for the first time the open source code PROPhet (short for PROPerty Prophet), which was developed to help address these challenges. PROPhet, which couples directly to the first-principles codes VASP^8–10^, Quantum Espresso^11^, and FHI-Aims^12, 13^, allows users to employ neural networks to fit a set of virtually any system properties (including scalar, vector, and/or grid-based quantities) to any other. PROPhet can be used, as artificial neural networks (ANNs) have in the past, to create analytical potentials^14–16^ or to predict properties based on selected descriptors^17–20^. However, its capabilities go much further, enabling the generation of density functionals for virtually any system property. Such functionals can greatly reduce the computational cost associated with the calculation of system properties. These functionals themselves are not limited to the familiar exchange-correlation functionals, but can be functionals for any number of complex properties such as electronic kinetic energy, or even more esoteric functionals (guaranteed to exist by the Hohenberg-Kohn theorem^1^) for system properties such as band gap. The comparatively low cost of evaluating neural networks means that such functionals would allow quantum mechanical methods to be applied to much larger systems than is possible with direct *ab initio* approaches. This paper serves as both a proof-of-concept exploring these more exotic uses of ANNs within chemical physics and as a first description of PROPhet, a tool created to facilitate further community exploration of these ideas.

In the following, these methods will be explored through a series of examples. We first discuss the creation of an analytical potential for diamond-phase carbon that includes vacancy defects, demonstrating PROPhets powerful capability to generate and explore the properties of complex energy surfaces. ANNs enable users to train potentials on small systems within the reach of *ab initio* approaches, then use these potentials to investigate large, complex systems such as interfaces, surfaces, grain boundaries, and amorphous materials using, e.g., classical Monte Carlo (MC) and molecular dynamics (MD) simulations. While using ANN potentials for this purpose is not a new capability, PROPhet is the first freely available, open-source code for training ANN potentials that also enables direct application of the ANN potentials within the widely used MD code, LAMMPS^21, 22^. It is also closely integrated to the first-principles DFT codes Quantum Espresso^11^, VASP^8–10^, and FHI-Aims^12, 13^, making it easily accessible for use by a large community of researchers. As an example of this approach, we use our diamond potential to compute the speed of sound in diamond as a function of carbon vacancy concentration in a regime of dilute vacancies that is inaccessible with DFT. Second, using gas phase NH_3_ as an example system, we demonstrate the ability of PROPhet to find exchange-correlation density functionals. We show that it is possible to map fully local functionals of the DFT charge density to the exchange correlation energy computed using either of two highly computationally expensive methods: DFT with the non-local B3LYP functional and CCSD(T). Further, we show that the predictions from the learned density functionals have near chemical accuracy with respect to the B3LYP and CCSD(T) values. Finally, we demonstrate the use of PROPhet to explore charge density functionals for properties other than energy, identifying a functional of the charge density that predicts, with high accuracy, the HOMO-LUMO gap of NH_3_ as computed within the expensive yet highly accurate G_0_W_0_ approximation. These examples provide a brief glimpse of the range of possibilities for using the approaches within PROPhet to develop fundamental chemical and physical models and to improve existing *ab initio* methods.

## Methods

### Overview

PROPhet was designed to be used primarily by researchers in the physics, chemistry, and materials science fields who are interested in using the power of machine learning approaches to extend the impact and applicability of first-principles computations. As some novel approaches are used by PROPhet (particularly with regard to providing complex system properties as input to neural networks), researchers in the machine learning community may also be interested in this work. In this section, we provide an overview of the underlying machine learning methods employed within PROPhet; these ideas are explored in more detail through a series of examples in the following sections.

At its core, PROPhet utilizes traditional fully connected, feed-forward neural networks. Training of the network parameters is performed via well known steepest-descent methods or more cutting edge methods such as resilient backpropagation^23^ (Rprop) and limited-memory BFGS^24^ algorithms. The code allows users to specify many details about the training, including the structure of the network, the type of transfer functions, the type of training algorithms, use of regularization, etc. Despite this flexibility, the aim of this work is not focused on the active development of the underlying machine learning approaches, but rather on their application to the *ab initio* field. To this end, PROPhet was designed for ease of use by non-experts in machine learning, implementing sensible defaults for most parameters. For the same reason, PROPhet is built to interface with several widely used first-principles codes (currently Quantum Espresso^11^, VASP^8–10^, and FHI-Aims (FHI)^12, 13^), allowing it to read data directly from their output files. A plugin mechanism makes adding new interfaces relatively easy, and users are encouraged to contribute an interface for their favorite first-principles code.

### ANN potentials

One of the key features of PROPhet is the ability to use the arrangement of atoms as the input to a neural network (NN), enabling one to identify structure-property relationships for a wide range of properties. When the output property is the total energy, the trained NN is an analytical potential that can be used within classical MC and MD simulations to study structural, thermal, and mechanical properties of systems with tens of thousands of atoms (well beyond the practical reach of direct *ab initio* computations) with the accuracy of DFT, or whatever method is used to generate the properties to which the NN is trained. To facilitate this, PROPhet is built with a library allowing the use of potentials created in PROPhet to be used directly in the LAMMPS MD package.

Finding the most effective way to use atomic structures as the inputs to a NN remains an active area of research within the community. PROPhet employs one of the most commonly used approaches, the Behler-Parrinello method, which is described in detail in refs 14, 25 and 26. Briefly, this method assigns a unique neural network to each species of atom in the system, rather than a single network for the whole system. This approach is advantageous in that the complexity of the training scales with the number of species rather than the total number of atoms. Mapping functions convert the atomic structure within a chosen cutoff radius around each atom to a vector of network inputs, which are a fingerprint of the chemical environment of that atom. The mapping functions also serve to automatically handle symmetries, including permutation symmetry, without the need for complex user tuning of basis functions or any special knowledge of the system.

In the case of a NN potential, when the network is evaluated, each network provides the contribution to the total energy from a single atom. The atoms are then looped over, with the appropriate species network evaluated for each one; the sum of their outputs is then the total energy of the system. This approach has several advantages over other methods^14, 27^. Most notably, since the inputs to the network depend only on the local environment around each atom, systems of many different sizes can be used in training and evaluation. As an example, this enables the creation of a training set that includes the atomic structures of small, representative bulk crystal supercells, bulk liquid phases, surfaces, and model solid-liquid interfaces to create a NN potential that can be used to investigate the properties of nanostructures in an explicit aqueous environment, which requires thousands of atoms and cannot be studied directly using DFT (e.g. see refs 28–30). One could also train on a set of bulk structures with varying supercell size and defect concentration to develop a NN potential capable of studying large bulk systems with dilute defect concentrations that are inaccessible to DFT; an example of this in the carbon system is discussed below.

We note that the range of applicability of a given NN potential is highly dependent on the input data set and should always be validated carefully.

### Charge density functionals

One of the novel capabilities of PROPhet is the ability to train charge density functionals to arbitrary system properties. According to the Hohenberg-Kohn theorems, the ground-state charge density is a fundamental variable that can be used to determine *any* system property, if only the means to extract the information is known^1^. One can write a reasonably general functional of the density as

1 $$\begin{matrix}{\rm{\Theta }}[\rho (\vec{r})]=\int f(\rho ({\vec{x}}_{1}),\rho ({\vec{x}}_{2}),\cdots ,\rho ({\vec{x}}_{n})){d}^{n}V\end{matrix}$$

where *n* is the order of the functional (*n* = 1 corresponds to a local functional) and *V* is the relevant region of the density. As a familiar concrete example, consider the local density approximation (LDA), where one writes

2 $$\begin{matrix}{E}_{xc}^{LDA}[\rho (\vec{r})]=\int \rho (\vec{r}){\varepsilon }_{xc}(\rho (\vec{r}))d\vec{r}\end{matrix}$$

where *ε*_*xc*_ is the so called exchange-correlation energy density, the form of which is known in the high and low density limits, and can be numerically interpolated for intermediate densities. Equation 2 is a special case of Equation 1 for which *n* = 1 and *f*(*ρ*) = *ρε*_*xc*_(*ρ*). Outside of the homogeneous electron gas, and for virtually all properties, Θ, other than energies, the function *f* is completely unknown *a priori*. Again, one of the strengths of machine learning techniques such as neural networks is that no *a priori* knowledge of the functional form is required, and the flexibility of the networks allows many complicated functions to be fit.

Fitting ${\rm{\Theta }}[\rho (\vec{r})]$ directly with a neural network is not feasible, since a neural network must always see the same number of inputs, each of which must have the same meaning, but a charge density represented on a grid, or expanded in some basis, can comprise an arbitrary and *a priori* unknown number of inputs. Worse, if the system of interest is shifted or rotated relative to the underlying grid or coordinate system, the resulting input vector presented to the network will change. Without employing a substantially expanded training set or explicitly symmetrizing the inputs in a preprocessing step, a neural network fit in this way will yield inconsistent answers. In addition, the number of parameters in a neural network grows quickly with the number of inputs. Given that the number of grid points or expansion coefficients for a charge density can easily exceed 10^6^, tens or hundreds of millions of DFT calculations might be required to provide enough training data.

PROPhet solves these problems by training the ANN as the kernal function *f* (i.e., the integrand) in Equation 1, rather than the entire functional, Θ. That is, the ANN itself is a pure *function* of the density — taking exactly *n* points as input (one for a local functional or a few for a nonlocal functional) per system, regardless of the number of grid points in the density. This network-represented function is then numerically integrated explicitly to form the full density *functional*. Since the number of inputs remains constant and relatively small regardless of the size of the grid, functionals created in PROPhet can be used on densities of any size without the need for preprocessing or using an excessive training data set. In addition to simplifying the evaluation of the functionals, this makes it possible to create a single functional applicable to many different physical systems, since the details of the underlying charge density grids need not be the same. Furthermore, density functionals written in this way return consistent results even if the physical system is shifted or rotated relative to the underlying coordinate system. Such transformations do not change the points the network sees, but only (possibly) the order in which it sees them, which does not affect the result of Equation 1. In PROPhet, the integration in Equation 1 is carried out by performing *n* nested loops over the density, evaluating the network at each point (or pair, triple, etc), and then summing these results after scaling by the appropriate *dV* value (the discrete volume). The result is the value of the functional Θ for the given density.

In order to train this density-functional network, we define an error function, *E*, as the sum of the squared errors over the training set, and determine how this error varies as each internal network parameter, *χ*_*i*_, changes. This is done by invoking the chain rule on Equation 1:

3 $$\frac{dE}{d{\chi }_{i}}=\frac{\partial E}{\partial {\rm{\Theta }}}\frac{\partial {\rm{\Theta }}}{\partial {\chi }_{i}}=\frac{\partial E}{\partial {\rm{\Theta }}}\int \frac{\partial f}{\partial {\chi }_{i}}{d}^{n}V.$$

In other words, we compute the derivative of the network output with respect to the internal parameters in the usual way, then integrate these derivatives scaled by $\frac{\partial E}{\partial {\rm{\Theta }}}$, the derivative of the error function with respect to the value of the functional, to get the derivatives required to properly adjust the parameters. The integration over volume evident in Equations 1 and 3 is the key difference between a standard implementation of ANNs and the way in which PROPhet handles functionals of grid-based data. For dense charge density grids, these integrals may entail a large number of points, greatly increasing the computational cost, especially for non-local functionals. For this reason, PROPhet allows for down-sampling, wherein density values are averaged over clusters of neighboring grid points. This effectively renormalizes the density to a coarser grid before the integrations are performed, greatly reducing the computational cost.

It is important to note that nothing in Equations 1 and 3 requires the functional being determined to represent an energy. Historically, the community has thought entirely in terms of energy functionals since we have some idea how to write approximations to these using known physics. However, the Hohenberg-Kohn theorems state that the ground-state charge density is a fundamental variable of the system, much like the full many-body wave function, and thus it contains the information necessary to determine all system properties. Below, we will provide an example illustrating how powerful ANNs can be for identifying density functionals to predict other properties.

## Results and Discussion

In this section, we present several examples to illustrate the broad and exciting range of applications that may be addressed using *ab initio* computations in conjunction with the machine learning tools developed in PROPhet.

To demonstrate the ability of PROPhet to map atomic structure and crystallographic information to system properties, we use the Behler-Parrinello approach described above to fit an analytical potential for bulk carbon in the diamond structure to DFT total energies. The fit was performed on around 28,000 (2 × 2 × 2) supercell structures (64 atoms each), generated using *ab initio* MD simulations at temperatures ranging from 50 K to 400 K. The DFT calculations were carried out within VASP, using a plane-wave cutoff of 350 eV, a (3 × 3 × 3) Γ-centered *k*-point grid, and the PBEsol exchange correlation functional. The ANN used to fit these data consisted of two hidden layers, each with 35 nodes (using a tanh transfer function) and a single linear activation node as the network output.

**Figure 1:**
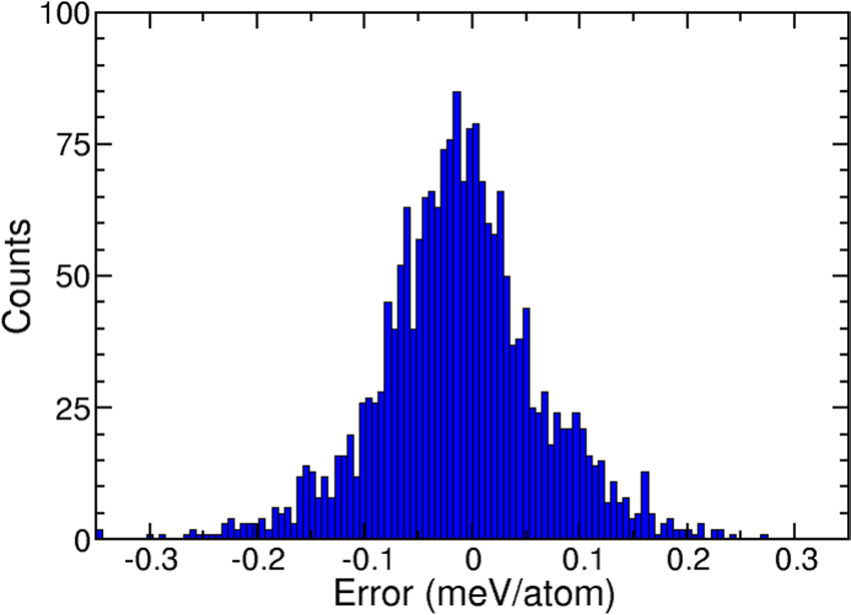
Histogram of the errors between a machine-learned analytical potential and the directly computed DFT energies for carbon in the diamond structure. The network used here, trained in PROPhet, contained 2 hidden layers each containing 35 nodes. The errors shown here are for 2000 structures that were not used in the fitting procedure.

**Figure 2:**
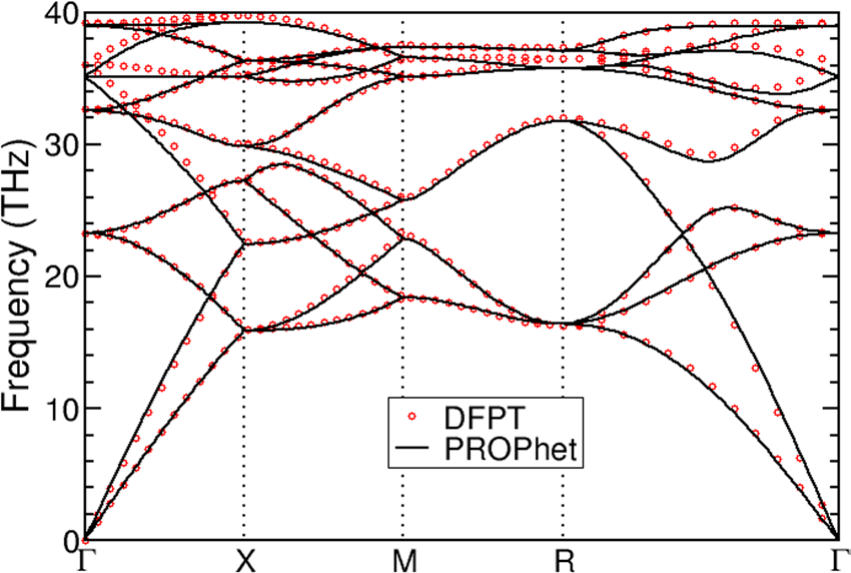
Phonon band structure of diamond-structured carbon as calculated from the PROPhet potential (black lines) and density functional perturbation theory (red circles). Phonons with the PROPhet potential were computed within the frozen-phonon approximation using a 6 × 6 × 6 supercell, while those from DFPT were computed within VASP using a primitive cell. All band structures were interpolated using the phonopy^43^ code. These results are in good agreement with experimental and other theoretical results^44, 45^.

Figure 1 shows a histogram of the prediction errors of the generated ANN potential over a testing set of 2000 structures not used in the fit. All errors are less than 0.5 meV per atom, well within the accuracy of the DFT calculations themselves. The quality of the fit can be further demonstrated by analyzing the phonon spectrum of a bulk-diamond supercell. Figure 2 compares the phonon band structure computed from this ANN potential for a (4 × 4 × 4) supercell with that computed using density functional perturbation theory within VASP. The agreement is excellent, bolstering confidence in the fidelity of the potential. Note that the neural network potential is orders-of-magnitude faster than the original DFT calculations, and the PROPhet phonon spectrum of Fig. 2 was computed in seconds.

The power of MD potentials generated from PROPhet is that they extend the accuracy of quantum mechanical methods for short-ranged interactions to very large systems with perhaps many thousands of atoms. This can be used to determine, e.g., thermal or mechanical properties, which are not easily accessible to direct *ab initio* calculations because of the prohibitive computational cost. As an example, the previously described diamond potential was extended to allow the study of dilute vacancies, by including a total of 75,000 structures, about half of which contained one or more vacancies. The data set was generated using *ab initio* MD in a manner identical to the above mentioned data set for ideal crystals. The resulting potential was then used to study the propagation speed of low-amplitude waves moving though diamond as a function of vacancy concentration. These calculations were carried out using the MD code LAMMPS^21, 22^, with a library created, and included within PROPhet, to allow potentials created with PROPhet to be used directly in LAMMPS as if they were native to the code.

The MD simulations were performed using a (6 × 6 × 10) supercell (2880 atoms). For each vacancy concentration considered, carbon atoms were removed at random, with care taken to avoid vacancy clusters since these are outside the range of validity of the generated potential. To investigate the effects of the precise vacancy configurations, 50 randomly generated structures were generated at each vacancy concentration. The simulations were conducted by first minimizing the energy for the given configuration, then applying a 4 Å/fs velocity to the atoms in the first plane, in either the *x* (transverse waves) or *z* (longitudinal waves) direction and monitoring the propagation of the impulse wave as it moves through the lattice.

**Figure 3:**
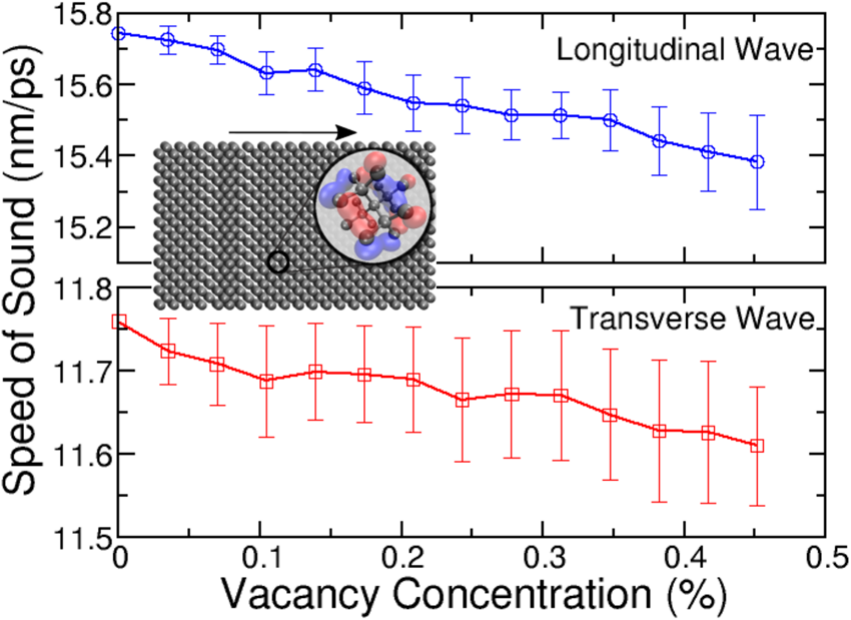
The speed of sound through a (6 × 6 × 10) unit cell of diamond as a function of vacancy concentration for both the longitudinal (top) and transverse (bottom) polarizations. The values and error bars plotted at each concentration correspond to the mean and standard deviation for the value of 50 randomly selected vacancy configurations. The inset shows a longitudinal wave propagating through the unit cell used for these calculations, and is meant to emphasize that potentials of this kind can couple short-range quantum mechanical information acquired from *ab initio* calculations with large, complex systems, facilitating the study of emergent behavior. The longitudinal speed of sound at zero vacancy concentration is in good agreement with experimental values^46^.

Figure 3 shows the calculated speed of sound averaged over all 50 randomly-generated vacancy configurations at each concentration, for longitudinal and transverse polarized waves moving through the cell. As is evident in the figure, the wave propagation speed generally decreases for both polarizations with increasing vacancy concentration, with the effect being larger for longitudinal waves. This stems from weaker coupling as vacancy concentration increases, creating a lower effective restoring force for atomic displacements. The increasing standard deviation (denoted in the figure by the error bars) as concentration increases arises from the local variation of wave propagation speed within the precise configuration of vacancies even at a given concentration. As the vacancy concentration increases, there are more possible configurations of vacancies and thus a larger range of possible wave propagation speeds. For example, at the highest concentration investigated (0.45%), the full range of wave propagation speeds over the 50 sampled configurations was 1129 m/s or about 7.35% of the average value. Such a wide variation means that a potential of this kind would be invaluable in studying, for example, thermoelectric materials for which one wishes to optimize the configuration of defects to minimize thermal conductivity (i.e., the propagation of phonons through the system). Note that these calculations could not be performed directly with DFT, since the low vacancy concentrations studied here require thousands of calculations on unit cells with hundreds to thousands of atoms, well outside the practical abilities of DFT. Further, results of this kind could not be achieved through traditional force-field type approaches since we are studying very precise vacancy configurations that *i*) cannot be modeled reliably by potentials fit to bulk system properties and *ii*) will tend to confound potentials based on connectivity. Through a combination of a robust set of DFT training data and a very flexible functional form, PROPhet is able to generate a near-DFT-accurate potential capable of studying such large-scale phenomena in systems with finely tuned microstructure.

Although creating analytical potentials capable of obtaining near-DFT energies orders-of-magnitude faster than *ab initio* methods is extremely useful, PROPhet’s capabilities extend well beyond this known use case. In particular, PROPhet can use the electronic charge density as input to a network, allowing one to train density functionals for arbitrary system properties. The most familiar example of such a use would be machine learning an exchange correlation energy functional. One way to proceed is to perform standard DFT calculations on many structures, extract the charge density from each calculation, then couple these to a more sophisticated DFT treatment (e.g., hybrid or meta-GGA functionals), or a wave function-based method (e.g., MP2 or CCSD(T)). Once trained successfully, the result is a pure density functional capable of obtaining (within some average error bars) energies from much more expensive, non-local approaches.

To demonstrate this, we first generate an exchange-correlation density functional for gas phase NH_3_, chosen because of the low computational cost of simulating such an atomic structure. Single point energy calculations for 1000 randomly-generated structures were carried out using the all electron FHI-Aims code^12, 13^ with the B3LYP^31^ hybrid functional using the “tight” basis set. The charge density for each structure, stored on an (80 × 80 × 80) grid, was used as input to a local PROPhet functional (Equation 1 with *n* = 1), with the output being trained to the B3LYP exchange-correlation energy. The chosen neural network contained a single hidden layer with 120 tanh-neurons. Errors for structures in a test set of 5000 structures not contained in the fit are impressively low, with an RMS error of only 20 meV, and almost all predictions lying within chemical accuracy of the underlying B3LYP values. It is critical to realize that these are the errors arising from using a pure (local) density functional to predict the value of a non-local hybrid functional. In effect, this functional approximates the B3LYP exchange-correlation energy, but at a computational cost similar to that of LDA.

An obvious question with these results is whether the relatively large number of parameters in the neural network (361 in this case) means that the good results are a matter of over-parameterization (this common criticism of neural networks conjures up quotes about fitting elephants^32^) or if real, meaningful information is being extracted from the density. To help answer this question, we performed a second fit using the same data, but with the exchange-correlation energies randomly reordered, so that each was associated with the “wrong” density. The neural network and the training procedure were otherwise identical to the previous case. The best RMS error achieved was 400 meV, a factor of 20 higher than that given above. This indicates that the previously fit network is indeed identifying information contained within the density that leads to the associated exchange-correlation energy. We emphasize that although the true exchange-correlation functional is universal, the functional fit here has extremely limited applicability as the input to the network is purely from NH_3_ data. A machine-learned functional with wider predictability would require a greatly expanded training set containing many structures from diverse systems. However, this demonstrates the promising ability of machine learning techniques to extract pure density functionals which closely approximate much more complicated wave function expressions.

**Figure 4:**
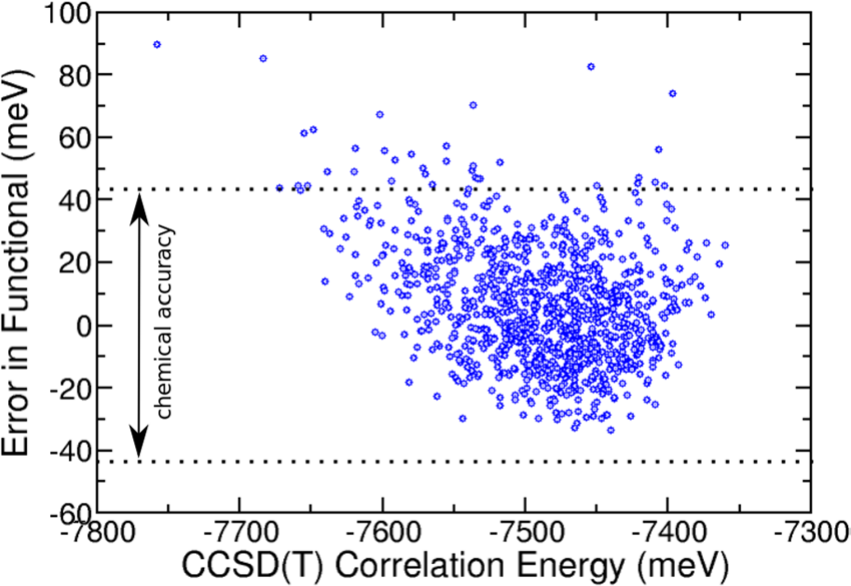
Accuracy of a PROPhet density functional mapping the Hartree-Fock electronic charge density to the CCSD(T) correlation energy. The underlying neural network consists of a single layer of 80 nodes and was trained on 500 randomly generated NH_3_ structures. The errors shown are relative to a CCSD(T) calculation carried out in NWchem for 1000 NH_3_ structures not contained in the fit. The dotted black lines denote chemical accuracy.

As a second, more intriguing example, a pure density functional was created to predict the CCSD(T) correlation energy of gas phase ammonia. The energies of 1500 randomly-generated NH_3_ structures were computed at the CCSD(T) level in the NWchem^33^ quantum chemistry package, using a cc-pVTZ basis set. Using, as input, the charge densities generated by the initial Hartree-Fock orbitals, 500 of these structures were used to train a PROPhet density functional consisting of a single layer with 80 neurons. The resulting density functional was then used to predict the CCSD(T) correlation energies of the remaining 1000 structures (see Fig. 4). As in the previous example, almost all the predictions lie within chemical accuracy of the reference method, demonstrating that this functional, again with a cost similar to an LDA calculation, can accurately predict the energy from much more accurate wave function approaches.

This ability to express non-local quantities, which depend explicitly on wave functions, as pure density functionals raises other intriguing possibilities. As an example, the well-known ${\mathscr{O}}({N}^{3})$ scaling of standard DFT limits its practical utility to systems of hundreds of atoms (with advanced methods reaching 1000 atoms). This steep scaling arises from the need to diagonalize the Hamiltonian to obtain the single-particle orbitals necessary to compute the electronic kinetic energy, which has no known pure density functional form. If a pure density functional (local or otherwise) can be found, the resulting *orbital-free* DFT could be capable of describing systems with tens of thousands of atoms^34^. The obvious implications of this have led to enthusiastic efforts to find such a pure density functional, an endeavor that dates back at least as far as the Thomas-Fermi approximation. While progress is being made in this field, to date no such functional has provided the necessary accuracy to supplant the current (exact) orbital-based calculation of the kinetic energy.

Recent work has shown machine learning to be a promising avenue toward finding a kinetic energy density functional^35–39^. In principle, the approach here is straightforward. Since any Kohn-Sham DFT calculation produces the exact (single particle) kinetic energy, one can run many single-point DFT calculations, extracting the charge density and kinetic energy from each. Equations 1 and 3 can then be used to fit the former to the latter, producing a kinetic energy density functional in the form of a trained ANN. In practice, of course, the complexity of such a kinetic energy density functional makes this a difficult process. As a demonstration of this concept, 500 NH_3_ structures were used as a training set to fit charge density to the electronic kinetic energy. The results, while not yet quantitatively sufficient, are encouraging for further exploration as a way to cheaply calculate the kinetic energy term. In a straightforward application of the approach, a two layer network with 25 tanh-neurons per layer yielded an RMS error of 300 meV over the same 5000 structure prediction set used above. While this is far from chemically accurate, it is important to note that even this first attempt exceeds, by orders of magnitude, the accuracy of the Thomas-Fermi model including the empirically scaled von Weizsäcker correction^40, 41^, which yields an RMS error of 450 eV over this set. It remains to be seen whether a carefully constructed network could outperform existing empirical results to an accuracy not currently feasible. A good approximation to the kinetic energy is likely to require a functional far more complicated than the purely local functional that we have employed. Work is currently underway to investigate the ability of non-local functionals, fit through PROPhet, to provide an improved description.

**Figure 5:**
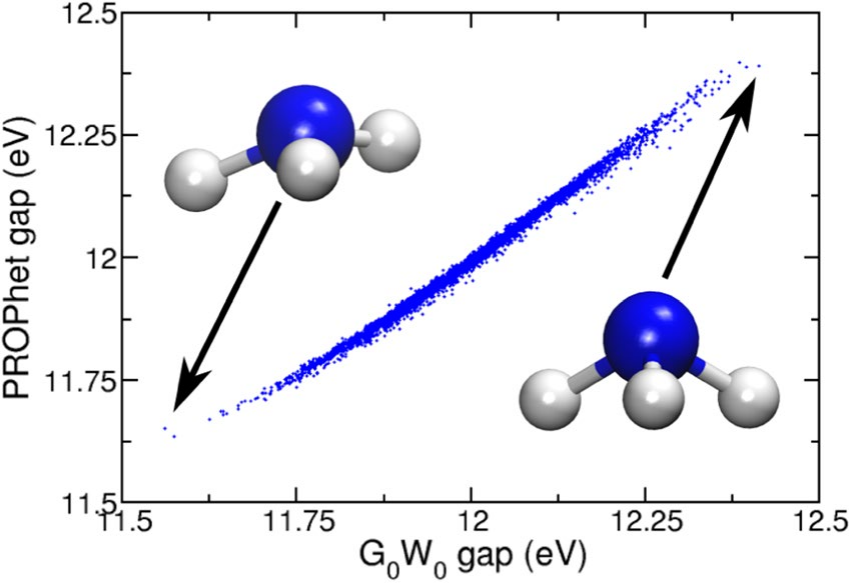
The HOMO-LUMO gap of 5000 structures of NH_3_ as computed via a functional of the total potential vs the same value computed directly within the G_0_W_0_ method. None of the structures in this figure were used in the fitting of the functional.

While a promising scientific endeavor, energy components are not the only useful quantities to compute using these methods. For example, optical properties such as band gap can be computed fairly accurately with advanced methods such as GW^3^ or TD-DFT^42^, but these accurate methods come with a substantial computational cost. This is not a significant problem if one is interested in only a few simple systems, but can become prohibitive if one is interested in complex system (e.g., interfaces) or needs to investigate many system (as might be the case during high-throughput searches for improved photo-absorber materials). In these cases, one is generally forced to use less accurate approximations to the band gap, which may lead to incorrect results. Extending the ideas described thus far, it is easy to see how an approximation to accurate band gaps can be generated with PROPhet. To demonstrate this, we again use the NH_3_ data to fit a charge-density functional to the HOMO-LUMO gap computed within the G_0_W_0_ approximation. In other words, we are mapping B3LYP charge density to the highly accurate G_0_W_0_ band gap. A network with 120 tanh-neurons in a single hidden layer produced the results shown in Fig. 5. The RMS error of this functional over 5000 structures not contained in the fit is a mere 10.4 meV, indicating an exceptionally accurate approximation to the gap computed with G_0_W_0_. Functionals like this could be used to post-process a standard DFT calculation and provide an excellent approximation to what one would obtain from a vastly more expensive GW calculation. Note that, while we have used simple G_0_W_0_ values here as our training targets, there is no reason one could not use fully self-consistent GW, TD-DFT, or any other desirable method to train the functional. With this approach, the expense of the underlying reference method only needs to be incurred while preparing the training set. After training, evaluating the functional to predict new values is of negligible computational cost.

These results demonstrate the range of possibilities of PROPhet and the novel ability to train charge density functionals in a machine learning framework. While powerful, the limitations of this method are not currently known and is an active area of research. As with all machine learning methods, this suite of tools is ultimately limited by the quality and quantity of the data in the training set. While there are significant computational advantages to the methods we have outlined, the computational cost associated with generating a representative dataset may be prohibitive for some properties or systems. Even with these limitations, however, the tools within PROPhet may be applied to a wide range of areas in physics, chemistry, and materials science.

## Conclusions

The results described here demonstrate great promise for using machine learning techniques to find less expensive alternatives for computing molecular and materials properties. While some of these ideas (e.g., analytical potentials) have been used successfully for quite some time, our results confirm that machine learning techniques can have a far wider impact if coupled to *ab initio* computations effectively, particularly by creating density functionals for important system properties. With the hope that this potential can be realized through community effort, we provide PROPhet as an open source tool to facilitate work in this direction. As described in this work, the current capabilities of PROPhet already allow a diverse array of uses, but the code is being released open source to allow its capabilities to be extended as needed by any interested researchers.

## Associated Content

### Supporting Information

A full detailed description of PROPhet, as well as the code itself can be found at http://kolpak.mit.edu/PROPhet.
